# Worklife expectancy in a cohort of Danish employees aged 55–65 years - comparing a multi-state Cox proportional hazard approach with conventional multi-state life tables

**DOI:** 10.1186/s12889-017-4890-7

**Published:** 2017-11-15

**Authors:** Jacob Pedersen, Jakob Bue Bjorner

**Affiliations:** 1 National Research Centre for the Working Environment (NRCWE), Lersø Parkallé 105, 2100 Copenhagen Ø, Denmark; 2 Department of Public Health, University of Copenhagen, Copenhagen, Denmark; 3 Optum, Johnston, RI USA

## Abstract

**Background:**

Work life expectancy (WLE) expresses the expected time a person will remain in the labor market until he or she retires. This paper compares a life table approach to estimating WLE to an approach based on multi-state proportional hazards models. The two methods are used to estimate WLE in Danish members and non-members of an early retirement pensioning (ERP) scheme according to levels of health.

**Methods:**

In 2008, data on self-rated health (SRH) was collected from 5212 employees 55–65 years of age. Data on previous and subsequent long-term sickness absence, unemployment, returning to work, and disability pension was collected from national registers. WLE was estimated from multi-state life tables and through multi-state models.

**Results:**

Results from the multi-state model approach agreed with the life table approach but provided narrower confidence intervals for small groups. The shortest WLE was seen for employees with poor SRH and ERP membership while the longest WLE was seen for those with good SRH and no ERP membership. Employees aged 55–56 years with poor SRH but no ERP membership had shorter WLE than employees with good SRH and ERP membership. Relative WLE reversed for the two groups after age 57.

At age 55, employees with poor SRH could be expected to spend approximately 12 months on long-term sick leave and 9–10 months unemployed before they retired – regardless of ERP membership. ERP members with poor SRH could be expected to spend 4.6 years working, while non-members could be expected to spend 7.1 years working.

**Conclusion:**

WLE estimated through multi-state models provided an effective way to summarize complex data on labor market affiliation. WLE differed noticeably between members and non-members of the ERP scheme. It has been hypothesized that while ERP membership would prompt some employees to retire earlier than they would have done otherwise, this effect would be partly offset by reduced time spent on long-term sick leave or unemployment. Our data showed no indication of such an effect, but this could be due to residual confounding and self-selection of people with poor health into the ERP scheme.

## Background

In European countries, the relative high income taxes finance the social welfare and secure social benefits for the citizens if they become sick-listed, unemployed, or if they retire early [1]. However, the aging workforce in most high income countries poses a threat to labor market participation and therefore severely challenges the social welfare systems. In Denmark, the government has taken several initiatives to maintain high labor market participation:

1. Limiting access to social benefits and reducing the maximum duration of time in which the social benefit can be received. For example, the length of time the employer is responsible for sickness compensation was increased from 15 days in April 2007 to 21 days in June 2008 and subsequently to 30 days in January 2012. Also, the duration of high level unemployment benefits was reduced from four to 2 years in October 2010 (www.retsinformation.dk).
2. Increasing the official retirement age from 65 to 67 for people born after 30 June 1960, and planning to increase it further if the average life expectancy increases (www.retsinformation.dk).

To evaluate the impact of such policies, statistical models need to handle multiple potential outcomes. For example, increasing the retirement age may cause employees in poor health to use long-term sick leave, thus not leading to the expected gains in total productivity. Evaluation of impact – and the statistical modeling hereof – needs to consider work, sickness absence, unemployment, return to work, and early retirement.

A newly developed method for analyzing labor market affiliation by the use of multi-state analysis on register data has shown advantages over traditional analysis in dealing with this complexity (www.retsinformation.dk) [2–5]. However, while multi-state models can provide a very detailed analysis, the results are complex and it can often be useful to combine the results into simpler statistics. Work life expectancy (WLE) may be such a simple statistic. For a given age, WLE states the expected number of years of labor market affiliation until official retirement age. The WLE is defined by the expected time a person will remain in the labor market until he or she retires. The flexibility of the Danish labor market implies that WLE cannot solely be defined by time in work as employees can be expected to spend some time in sickness absence or unemployment. Thus, the labor market affiliation should be measured by time spent in any of these three states although the time spent in each state can be separated.

Methods for estimating WLE have gained increasing attention through the last decade. The standard method for determining WLE is the Markov increment model (MID) [6, 7]. Researchers have suggested [7] using the multi-state life table (MSLT) method to estimate WLE [8, 9]. The MSLT method is based on more detailed estimations of transitions probabilities than the MID method, and can handle multiple events in discrete time. However, concerns may be raised that the use of the MSLT method may lead to unstable WLE estimates for small subgroups due to the lack of sufficient numbers of events. The concern is related to the nature of the method, as it relies on estimating nonparametric transition intensities for calculating WLE.

By using a multi-state model that includes all relevant states, it is possible to achieve detailed estimates on the years spent in each state and to summarize the estimate as WLE. A multi-state model (eg, based on the proportional hazards (Cox) model) can estimate the transitions intensities even for small groups by utilizing the estimated hazard ratios, the estimated baseline hazard, and the proportional hazards assumption [10]. This model can also be used to estimate the impact of an intervention on WLE [2]. This is done by comparing the expected duration of time spent in each state between the group receiving the intervention and the group not receiving the intervention.

The purpose of the present article is to compare a new multi-state Cox-regression method for estimation WLE, with the conventional MSLT method. The multi-state Cox-regression method can be interpreted as a baseline MSLT adjusted with estimates from a Cox-regression model (Cox-MSLT). We conduct the method comparison though an example where we study the effect on WLE of poor health and the financial possibilities for early retirement. In addition to WLE, we study the expected time spent in long-term sickness absence and in unemployment. To provide perspective on the analyses, we provide a short summary of labor market conditions in Denmark and a discussion of statistical approaches to estimating WLE.

### The Danish labor market system

The Danish labor market can be described as a flexicurity system with high labor market participation rates, low formal employment protection, generous and accessible social benefits, and a high turnover of the work force between employments [11]. Among the Danish social benefits are two early retirement schemes (the voluntary early retirement pension (ERP) and the disability pension scheme), sickness absence benefits, and unemployment benefits. All these benefits are registered in databases maintained by Statistics Denmark.

In the ERP scheme, the employee pays a monthly fee to qualify for early retirement. The ERP is co-financed by the state. Until 2014, the employee was qualified for ERP at the age of 60 if he or she had paid into the scheme for 30 years and was available for work. The employee could achieve a higher ERP compensation by postponing retirement until the age of 62. ERP payments stop at the standard retirement age (currently 65) and individuals shift to a state pension.

The disability pension scheme is open to all Danish residents with limited workability, irrespective of a preceding career on the labor market. The Danish system contains several types of disability pensions, and some also contain a certain amount of labor market affiliation (eg, the flex-job scheme).

In general, employees receive a salary when sick-listed. Typically, the expenses are paid by the employer as normal salary, but for long-term sickness (in 2008: longer than three consecutive weeks), some of the expenses for salary are reimbursed by the municipality. If a sick-listed person becomes unemployed, then the municipality will be paying the sickness absence benefit directly to the sick-listed person. Special arrangements are available for certain groups, eg, people with chronic disease for which insurance can be established, allowing the employer to obtain reimbursement from day one of sickness absence (the so-called §56 scheme (www.retsinformation.dk)).

In case of unemployment, members of an insurance fund receive unemployment benefits, if they are available for the labor market. People with no membership of an insurance fund may qualify for social assistance benefits, depending on the total household income.

## Methods

### Estimating WLE through multistate life tables and Cox models

**Fig. 1:**
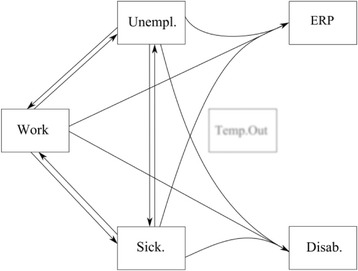
The Multi-state model with five primaries states; Work, Sickness absence, Unemployment, Disability pension, Early retirement pension scheme (ERP), and one secondary state (Temp. Out). The eight possible transitions are represented by *arrows*

The present paper uses a multi-state model representing the Danish labor market system by five primary states: working (*W*), sickness absence (*S*), unemployment (*U*), disability pension (*D*), and ERP (*E*). Two secondary states are also included: temporary out (*TO*) represents the time when a person is not in one of the primary states and not censored, and a death state (Death) if a person dies during follow-up. The multi-state model is shown in Fig. 1 where states are represented by boxes and the possible transitions are represented by arrows. The two states D and ERP are treated as absorbing states (as is the secondary Death state), meaning that if a respondent reaches either of these states, we assumed that no further transitioning is possible. The three primary states – *W, S and U* (and the secondary state *TO*) – are treated as transdurable states which mean that recurrent events are possible. The individual states are explained in detail under the title “Classification of the states in the multi-state model” in the “Data” section. WLE is estimated on the basis of the multi-state model for any age above 55 years and to the pension age of 65 years. People are being censored when turning 65, or if they reach the end of the follow-up period.

Due to sparse data, we were not able to estimate the risks of either D or ERP separately from work, sickness absence and unemployment. Therefore, we assume a combined risk of disability pension from each of these three states. Similarly, we had to assume a combined probability of ERP across these three states.

The estimated WLE is based on the estimation of the intensity matrix A(t) (sometimes also called the time dependent instantaneous transition matrix). For a particular timepoint (t), the intensity matrix shows for each initial state *h* the instantaneous probability of transitioning to another state *j* (this probability is called the state-specific instantaneous transition probability *α*_*hj*_(*t*)) as well as the probability of staying in the same state (called the state-specific intensity *α*_*h*_(*t*)). The MSLT method uses a direct calculation of the intensity matrix whereas the COX-MSLT method implies an estimation of the intensity matrix based on the baseline hazard, which then can be adjusted by parameters estimated by the Cox-analysis.

To estimate the instantaneous transition-specific intensities for the MSLT method, one would use equation (1) in which *d*_*hj*_(*t*) is the number of transitions from state *h* to state *j* at time (*t*), *n*_*h*_ is the number individuals at risk of the transition, located in state *h* just before time (*t*):

1 $$ {\widehat{\boldsymbol{\upalpha}}}_{\mathbf{\mathsf{h}\mathsf{j}}}\left(\mathbf{\mathsf{t}}\right)=\frac{{\mathbf{\mathsf{d}}}_{\mathbf{\mathsf{h}\mathsf{j}}}\left(\mathbf{\mathsf{t}}\right)}{{\mathbf{\mathsf{n}}}_{\mathbf{\mathsf{h}}}\left(\mathbf{\mathsf{t}}\right)} $$

The state-specific intensities are estimated by equation (2) in which *m* is the number of states and *k ≠ h*:

2 $$ {\widehat{\boldsymbol{\upalpha}}}_{\mathbf{\mathsf{h}}}\left(\mathbf{\mathsf{t}}\right)=-\frac{\sum \limits_{\mathbf{\mathsf{k}}=\mathsf{1}}^{\mathbf{\mathsf{m}}}{\mathbf{\mathsf{d}}}_{\mathbf{\mathsf{h}\mathsf{k}}}\left(\mathbf{\mathsf{t}}\right)}{{\mathbf{\mathsf{n}}}_{\mathbf{\mathsf{h}}}\left(\mathbf{\mathsf{t}}\right)} $$

To estimate the intensity matrix using the Cox-MSLT approach, the multi-state Cox model must first be estimated. The Cox proportional hazards model (Cox model) for the occurrence of a transition can be specified using the intensity or hazard function *λ*(*t*) depending on the event history up to time (*t*). This function specifies that the risk of a transition in the interval from *t* to *t* + *h* is *λ*(*t*) ∙ *h* if the person is at risk just before time (*t*). Thus, the multi-state model is defined by transition intensity *λ*_*hj*_(*t*), which can be interpreted as the instantaneous probability of a specific transition from the state *h* to the state *j* at time (*t*)(in which : *h*, *j* ∈ (*W*, *S*, *U*, *D*, *ERP*)):

3 $$ {\boldsymbol{\uplambda}}_{\mathbf{\mathsf{hj}}}\left(\mathbf{\mathsf{t}}\right)={\mathbf{\mathsf{u}}}_{\mathbf{\mathsf{i}}}{\boldsymbol{\uplambda}}_{\mathbf{\mathsf{hj}},\mathsf{0}}\left(\mathbf{\mathsf{t}}\right)\mathbf{\mathsf{\exp}}\left({\boldsymbol{\upbeta}}_{\mathsf{1}}{\mathbf{\mathsf{Z}}}_{\mathsf{1}}\left(\mathbf{\mathsf{t}}\right)+\cdots +{\boldsymbol{\upbeta}}_{\mathbf{\mathsf{m}}}{\mathbf{\mathsf{Z}}}_{\mathbf{\mathsf{m}}}\left(\mathbf{\mathsf{t}}\right)+{\boldsymbol{\upbeta}}_{\mathbf{\mathsf{m}}+\mathsf{1}}{\mathbf{\mathsf{Z}}}_{\mathbf{\mathsf{m}}+\mathsf{1}}+\cdots +{\boldsymbol{\upbeta}}_{\mathbf{\mathsf{k}}}{\mathbf{\mathsf{Z}}}_{\mathbf{\mathsf{k}}}\right) $$

The baseline hazard *λ*_*hj*, 0_(*t*) is allowed to vary freely, and the coefficients *β* show the effects of the covariates *Z*. The model includes both time-varying covariates (*Z*(*t*)_1⋯*m*_), such as shifts between age groups, and time-constant covariates (*Z*_(*m* + 1)⋯*k*_) (eg, gender). The frailty term *u*_*i*_ is a random effect for each individual, assumed to be gamma distributed. The model assumes proportionality on each of the covariates (*Z*_(*m* + 1)⋯*k*_),and(*Z*(*t*)_1⋯*m*_) . This assumption can be evaluated by stratified cumulative hazards charts. Many potential time scales may be used: eg, calendar time or time since last transition. In this paper, the time scale *t* is age.

By arranging the data in a long format it is possible to analyze the entire multi-state model by one Cox-regression stratified by the transitions. The same estimates are achieved by arranging the data in a short format and then analysing each transition by a separate Cox-regression [12, 13].

The instantaneous transition-specific probabilities at time (*t*), are calculated by the slope of the transition specific cumulative hazard ${\widehat{\Lambda}}_{\mathrm{hj},0}\left(\mathrm{t}\right)$ from the SAS PHREG procedure [14]:

4 $$ {\widehat{\boldsymbol{\upalpha}}}_{\mathbf{\mathsf{hj}}}\left(\mathbf{\mathsf{t}}\right)=\frac{\mathbf{\mathsf{d}}{\widehat{\boldsymbol{\Lambda}}}_{\mathbf{\mathsf{hj}},\mathsf{0}}\left(\mathbf{\mathsf{t}}\right)}{\mathbf{\mathsf{d}\mathsf{t}}} $$

The state instantaneous intensity is estimated by:

5 $$ {\widehat{\boldsymbol{\upalpha}}}_{\mathbf{\mathsf{h}}}\left(\mathbf{\mathsf{t}}\right)=-{\sum}_{\mathbf{\mathsf{k}}=\mathsf{1}}^{\mathbf{\mathsf{m}}}{\widehat{\boldsymbol{\upalpha}}}_{\mathbf{\mathsf{h}\mathsf{k}}}\left(\mathbf{\mathsf{t}}\right) $$

The intensity matrix A(t) for the five primary states is shown in equation (6). The raw matrix is shown to the left and the final matrix representing combined transitions and absorbing states is shown to the right.

6 $$ \widehat{\mathbf{A}}\left(\mathbf{t}\right)={\left[\begin{array}{ccccc}\frac{-{\mathbf{d}}_{\mathbf{W}}}{{\mathbf{n}}_{\mathbf{W}}}& \frac{{\mathbf{d}}_{\mathbf{W}\to \mathbf{S}}}{{\mathbf{n}}_{\mathbf{W}}}& \frac{{\mathbf{d}}_{\mathbf{W}\to \mathbf{U}}}{{\mathbf{n}}_{\mathbf{W}}}& \frac{{\mathbf{d}}_{\mathbf{W}\to \mathbf{E}}}{{\mathbf{n}}_{\mathbf{W}}}& \frac{{\mathbf{d}}_{\mathbf{W}\to \mathbf{D}}}{{\mathbf{n}}_{\mathbf{W}}}\\ {}\frac{{\mathbf{d}}_{\mathbf{S}\to \mathbf{W}}}{{\mathbf{n}}_{\mathbf{S}}}& \frac{-{\mathbf{d}}_{\mathbf{S}}}{{\mathbf{n}}_{\mathbf{S}}}& \frac{{\mathbf{d}}_{\mathbf{S}\to \mathbf{U}}}{{\mathbf{n}}_{\mathbf{S}}}& \frac{{\mathbf{d}}_{\mathbf{S}\to \mathbf{E}}}{{\mathbf{n}}_{\mathbf{S}}}& \frac{{\mathbf{d}}_{\mathbf{S}\to \mathbf{D}}}{{\mathbf{n}}_{\mathbf{S}}}\\ {}\frac{{\mathbf{d}}_{\mathbf{U}\to \mathbf{W}}}{{\mathbf{n}}_{\mathbf{U}}}& \frac{{\mathbf{d}}_{\mathbf{U}\to \mathbf{S}}}{{\mathbf{n}}_{\mathbf{U}}}& \frac{-{\mathbf{d}}_{\mathbf{U}}}{{\mathbf{n}}_{\mathbf{U}}}& \frac{{\mathbf{d}}_{\mathbf{U}\to \mathbf{E}}}{{\mathbf{n}}_{\mathbf{U}}}& \frac{{\mathbf{d}}_{\mathbf{U}\to \mathbf{D}}}{{\mathbf{n}}_{\mathbf{U}}}\\ {}\frac{{\mathbf{d}}_{\mathbf{E}\to \mathbf{W}}}{{\mathbf{n}}_{\mathbf{E}}}& \frac{{\mathbf{d}}_{\mathbf{E}\to \mathbf{S}}}{{\mathbf{n}}_{\mathbf{E}}}& \frac{{\mathbf{d}}_{\mathbf{E}\to \mathbf{U}}}{{\mathbf{n}}_{\mathbf{E}}}& \frac{-{\mathbf{d}}_{\mathbf{E}}}{{\mathbf{n}}_{\mathbf{E}}}& \frac{{\mathbf{d}}_{\mathbf{E}\to \mathbf{D}}}{{\mathbf{n}}_{\mathbf{E}}}\\ {}\frac{{\mathbf{d}}_{\mathbf{D}\to \mathbf{W}}}{{\mathbf{n}}_{\mathbf{D}}}& \frac{{\mathbf{d}}_{\mathbf{D}\to \mathbf{S}}}{{\mathbf{n}}_{\mathbf{D}}}& \frac{{\mathbf{d}}_{\mathbf{D}\to \mathbf{U}}}{{\mathbf{n}}_{\mathbf{D}}}& \frac{{\mathbf{d}}_{\mathbf{D}\to \mathbf{E}}}{{\mathbf{n}}_{\mathbf{D}}}& \frac{-{\mathbf{d}}_{\mathbf{D}}}{{\mathbf{n}}_{\mathbf{D}}}\end{array}\right]}^{\mathbf{t}}\curvearrowright \widehat{\mathbf{A}}\left(\mathbf{t}\right)={\left[\begin{array}{ccccc}\frac{-{\mathbf{d}}_{\mathbf{W}}}{{\mathbf{n}}_{\mathbf{W}}}& \frac{{\mathbf{d}}_{\mathbf{W}\to \mathbf{S}}}{{\mathbf{n}}_{\mathbf{W}}}& \frac{{\mathbf{d}}_{\mathbf{W}\to \mathbf{U}}}{{\mathbf{n}}_{\mathbf{W}}}& \frac{{\mathbf{d}}_{\mathbf{W},\mathbf{S},\mathbf{U}\to \mathbf{E}}}{{\mathbf{n}}_{\mathbf{W},\mathbf{S},\mathbf{U}}}& \frac{{\mathbf{d}}_{\mathbf{W},\mathbf{S},\mathbf{U}\to \mathbf{D}}}{{\mathbf{n}}_{\mathbf{W},\mathbf{S},\mathbf{U}}}\\ {}\frac{{\mathbf{d}}_{\mathbf{S}\to \mathbf{W}}}{{\mathbf{n}}_{\mathbf{S}}}& \frac{-{\mathbf{d}}_{\mathbf{S}}}{{\mathbf{n}}_{\mathbf{S}}}& \frac{{\mathbf{d}}_{\mathbf{S}\to \mathbf{U}}}{{\mathbf{n}}_{\mathbf{S}}}& \frac{{\mathbf{d}}_{\mathbf{W},\mathbf{S},\mathbf{U}\to \mathbf{E}}}{{\mathbf{n}}_{\mathbf{W},\mathbf{S},\mathbf{U}}}& \frac{{\mathbf{d}}_{\mathbf{W},\mathbf{S},\mathbf{U}\to \mathbf{D}}}{{\mathbf{n}}_{\mathbf{W},\mathbf{S},\mathbf{U}}}\\ {}\frac{{\mathbf{d}}_{\mathbf{U}\to \mathbf{W}}}{{\mathbf{n}}_{\mathbf{U}}}& \frac{{\mathbf{d}}_{\mathbf{U}\to \mathbf{S}}}{{\mathbf{n}}_{\mathbf{U}}}& \frac{-{\mathbf{d}}_{\mathbf{U}}}{{\mathbf{n}}_{\mathbf{U}}}& \frac{{\mathbf{d}}_{\mathbf{W},\mathbf{S},\mathbf{U}\to \mathbf{E}}}{{\mathbf{n}}_{\mathbf{W},\mathbf{S},\mathbf{U}}}& \frac{{\mathbf{d}}_{\mathbf{W},\mathbf{S},\mathbf{U}\to \mathbf{D}}}{{\mathbf{n}}_{\mathbf{W},\mathbf{S},\mathbf{U}}}\\ {}\mathbf{0}& \mathbf{0}& \mathbf{0}& \mathbf{0}& \mathbf{0}\\ {}\mathbf{0}& \mathbf{0}& \mathbf{0}& \mathbf{0}& \mathbf{0}\end{array}\right]}^{\mathbf{t}} $$

Each element in the matrix represents a transition probability. For example, *d*_*W* → *S*_ is the number of transitions at time *t* from state *W* to *S* and *n*_*W*_ is the number of individuals at risk just before time *t* in state *W*. Since ERP (E) and D are considered absorbing states, transition probabilities out of these states are set to zero. The diagonal is estimated by the total number of transitions out of the particular state at time *t* multiplied by minus one, which corresponds to equation (2) for the MSLT method and equation (5) for the Cox-MSLT method.

The term *W,S,U* indicates the combined transition or risk set. The combined transitions imply a special case when the intensity matrix is produced, as the combined intensity must be redistributed between the origin transitions to make the intensity matrix valid. This issue has been overcome by redistributing the combined intensities according to crude frequencies of the origin transitions from before they were combined.

An intensity matrix is made for each event time, and the product integral formula is then used to estimate the transition probability matrices in the time span from *s* to *t* [15].

7 $$ \widehat{\mathbf{\mathsf{P}}}\left(\mathbf{\mathsf{s}},\mathbf{\mathsf{t}}\right)={\prod}_{\mathbf{\mathsf{s}}}^{\mathbf{\mathsf{t}}}\left(\mathbf{\mathsf{I}}+\widehat{\mathbf{\mathsf{A}}}\left(\mathbf{\mathsf{u}}\right)\right) $$

The diagonal element of the transition probability matrices expresses the state probability. The integral defined by the area under the state probabilities curves expresses the expected time spent in each state (in which : *h* = *j and h* = (*W*, *S or U*), and t = *t*_*pension*_).

8 $$ \mathbf{\mathsf{E}}\left(\mathbf{\mathsf{h}}\right)={\int}_{\mathbf{\mathsf{s}}}^{\mathbf{\mathsf{t}}}{\mathbf{\mathsf{P}}}_{\mathbf{\mathsf{h}\mathsf{j}}}\left(\mathbf{\mathsf{s}},\mathbf{\mathsf{t}}\right)\mathbf{\mathsf{du}} $$

By having a follow-up time that covers the time span from entry age (*s*) to pension age (*t*), it is possible to estimate the expected time spent in each state *E*(*h*) as the area under the state probability curve.

The integral can be calculated by the trapezium rule:

9 $$ \widehat{\mathbf{\mathsf{WLE}}}\approx \mathbf{\mathsf{\frac{1}{2}}}\widehat{\mathbf{\mathsf{P}}}\left(\mathbf{\mathsf{s}}\right)+\left(\;{\sum}_{\mathbf{\mathsf{k}}=\mathbf{\mathsf{s}}+\mathsf{1}}^{{\mathbf{\mathsf{t}}}_{\mathbf{\mathsf{p}}}-\mathsf{1}}\widehat{\mathbf{\mathsf{P}}}\left(\mathbf{\mathsf{k}},{\mathbf{\mathsf{t}}}_{\mathbf{\mathsf{p}}}\right)\right)+\mathbf{\mathsf{\frac{1}{2}}}\widehat{\mathbf{\mathsf{P}}}\left({\mathbf{\mathsf{t}}}_{\mathbf{\mathsf{p}}}\right) $$

WLE is then estimated by combining the expected time in each of the three states of; work, sickness absence, and unemployment.

Because the estimate of the expected time spent in each state is conditional on the starting state and the starting age, one can estimate a curve expressing the unconditional WLE for any age by making the same calculation for every possible starting age (*s*) until pension age (*t*).

The upper and lower bounds of the expected duration of years spent in either the work, sick-listed or the unemployment state is calculated separately. The lower bound is estimated as the area under the lower 95% confidence limits for the state probability, and the upper bound is estimated as the area under the upper 95% confidence limits for the state probability. The confidence interval for each state probability is estimated on the basis of data containing the transition specific risk-set, which is an additional optional output from the SAS PHREG procedure. The risk-set data is used to estimate the Greenwood variance for the empirical covariance matrix used in the recursion formula [16] explained in detail in the documentation for the etm and mstate package designed for the statistical software R [12, 17]. For the present study, the formula was recoded to the SAS software by the used of SAS HASH tables and multi-dimensional arrays.

When using the Cox-MSLT method, the WLE estimation can be conducted for any combination of covariates. This is done by using the estimates of the Cox-regression to adjust each element of the time dependent intensity matrices by equation (10) for the no diagonal elements.

10 $$ {\widehat{\upalpha}}_{\mathsf{hj}}\left(\mathsf{t}\right)={\widehat{\upalpha}}_{\mathsf{hj},\mathsf{0}}\left(\mathsf{t}\right)\mathsf{\exp}\left({\upbeta}_{\mathsf{1}}{\mathsf{Z}}_{\mathsf{1}}\left(\mathsf{t}\right)+\cdots +{\upbeta}_{\mathsf{m}}{\mathsf{Z}}_{\mathsf{m}}\left(\mathsf{t}\right)+{\upbeta}_{\mathsf{m}+\mathsf{1}}{\mathsf{Z}}_{\mathsf{m}+\mathsf{1}}+\cdots +{\upbeta}_{\mathsf{k}}{\mathsf{Z}}_{\mathsf{k}}\right) $$

By adjusting the intensity matrices it is possible to compare estimates of the WLE for different combinations of covariate (eg, different levels self-rated health). The multi-state design is typically following a Markov assumption, which means that a transition only depends on the current state and not on past transitions. However, because the present model includes variables indicating whether a person has previously experienced long-term sick-listing or/and unemployment periods during the follow-up period, the Markov assumption is violated and may cause biased results [4, 18, 19].

The predictive effect of the self-rated health is estimated by weighting the multi-state Cox-regression by “stabilized” inverse propensity scores regarding the probability of each health level as well as the probability of being right censored. The “stabilization” is done by multiplying the adjusted inverse propensity score by a non-adjusted propensity score. Because the analysis uses time dependent variables, a “stabilized” inverse propensity score of each level of self-rated health is calculated for each record in the data. Each “stabilized” inverse propensity score is in addition multiplied by the “stabilized” propensity score of being right censored. The “stabilized” inverse propensity scores were implemented by standard logistic regression in SAS, in accordance with suggestions by Hernán [20].

All analyses were done using SAS 9.4 (PROC Phreg, PROC Logistic, SAS HASH tables). The statistical software R has been used for checking the results of the matrix operations conducted in Base SAS using arrays. Multi-state calculation may also be conducted by the mstate package and the etm package for R [12, 17].

### Data

The Danish National Working Environment Survey (DANES) included a representative sample of 5212 members of the Danish working population in the age range from 55 to 64 years. The DANES study contains questions concerning health and the work environment collected in the years 2008 and 2009. The Danes survey includes three random subsamples with an overall response rate of 69%: 9913 persons aged 18–59 (response rate 66%), 4477 persons aged above 50 years (response rate 76%), and 3823 persons aged 18–59 and employed in one of 269 companies (response rate 68%). This sample was merged with data on death dates from Statistics Denmark and “The Danish Register of Sickness absence compensation benefits and Social transfer payments” (RSS) which is a national register containing registrations on all major social payments. The RSS contains extra details for registration of sickness absence benefit and maternity payments, and all such payment periods are registered by dates whereas all other benefits periods are registered in weeks.

All individuals entered the analysis at the date of returning the DANES questionnaire or when they turned 55. The cohort was followed in RSS in the years 2008 to 2013, which gives a follow-up time between four and 5 years per individual.

In the study sample, 77% were members of the ERP scheme, while the rest (23%) could only receive support for early retirement if they qualified for disability pension. All participants qualified for state pension at the official retirement age of 65 years.

### Covariates

The Cox-regression model included the following covariates; a yes/no variable obtained from the Statistics Denmark on individual ERP saving, gender, a yes/no variable for membership of a sickness insurance for individuals with chronic disease (§56), prior long-term sickness absence (LTS) (more than 4 weeks for 1 year before inclusion, or during follow-up) (yes/no), prior long-term unemployment (LTU) (more than 4 weeks for 1 year before inclusion, or during follow-up) (yes/no). The Cox-regression model was additionally adjusted for self-rated health which was included in the DANES by the question; “In general, would you say your health is? ,” with the responses “Excellent,” “Very good,” and “Good” indicating good health and the responses “Fair” and “Poor” indicating poor health. If a dynamic covariate shifted from “no” to “yes” during follow-up, the age was carried forward. This was also the case whenever a transition from one state to another occurred. The covariates were transition specific, so that each covariate could have different effects on different transitions.

For the MSLT model, the data was stratified by ERP scheme (yes/no) and self-rated health (good/poor). Thus, the MSLT chart is based on four separate analyses. Due to the small subsample size of particular the sample containing non ERP members with poor SRH, gender specific trajectories was not accommodated for. For the Cox-MSLT, the data was only stratified by ERP scheme. The Cox-MSLT charts were developed by adjusting the two baseline hazard curves for good self-rated health (member and non-members of the ERP scheme). The curves for poor self-rated health were calculated by adjusting the transition intensities by the corresponding estimates from the Cox regression.

### Classification of the states in the multi-state model

Separate analyses were conducted for people with the possibility of early retirement due to ERP and those without that possibility. In the latter analysis, the model was reduced to four primary states (W, S, U, and D), as ERP is not an option (the models also included the secondary TO state and the absorbing Death state). The work state contains all time periods when no social benefit payments are registered, (ie, time periods when the person is self-supporting or working) [21]. The sickness absence state is defined by receiving a sickness absence benefit for more than 3 weeks. The unemployment state is defined by reception of unemployment benefits or social assistance benefits. The disability pension state is defines by reception of disability benefit and the ERP state is defined by reception of ERP. Individuals granted disability pension benefits may still be available for the labor market or be employees, but only on special terms including benefits regarding: national supplementary disability pension (early retirement pension), light job, flexible job, or vacancy benefit for individuals with a flexible job. In this study, reception of any of these benefits was included in the definition of the disability pension state. All individuals were censored in the following situations; entering an absorbing state, at the end of the study period, or when they turn 65 years of age.

## Results

**Fig. 2:**
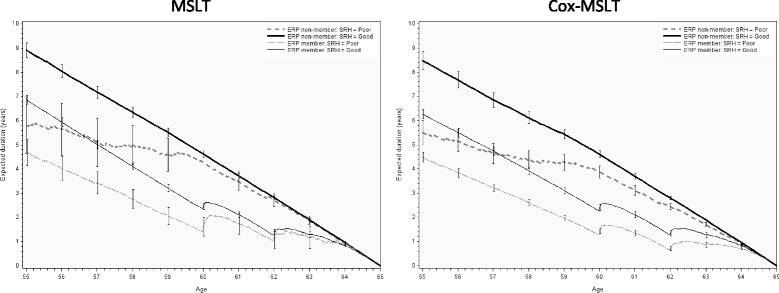
Work expectancy divided by self-rated health and ERP members and non-members: the MSLT in the *left* column, and the Cox-MSLT in the *right* column

The results of the comparison of the MSLT and the Cox-MSLT approach are found in Fig. 2. But the Cox-MSLT approach contains several intermediate steps of results, which are stated first.

Table 1 show that the cohort has a slight overweight of women having ERP membership. Almost 90% reported good self-rated health. Only 2% had the additional sick leave insurance for patients with chronical disease (§ 56), 15% had long-term sickness absence and 10% had registered unemployment in the year before baseline.

**Table 1:** The distribution of the population at the start of the follow-up period

		Non-member	ERP member
		N (%)	N (%)
	Total	1203 (23.08%)	4009 (76.92%)
Gender	Female	487 (40.48%)	2120 (52.88%)
	Male	716(59.52%)	1889 (47.12%)
Self-health	Good	1076 (89.44%)	3557 (88.73%)
	Poor	116 (9.64%)	415 (10.35%)
	NA	11 (0.91%)	37 (0.92%)
LTS	No	1032 (85.79%)	3384 (84.41%)
	Yes	171 (14.21%)	625 (15.59%)
LTU	No	1085 (90.19%)	3526 (87.95%)
	Yes	118 (9.81%)	483 (12.05%)
Chronic disease § 56	No	1186 (98.59%)	3912 (97.58%)
	Yes	17 (1.41%)	97 (2.42%)

*ERP* early retirement pension scheme, *LTS* long term sickness absence, *LTU* long term unemployment

During follow-up, almost 40% of ERP members had periods with sickness absence payment, compared to 34% of those without ERP membership (Table 2). The transition from sick-listing to work was only slightly smaller, indicating that almost all sick-listed individuals recovered. Slightly more than 46% of ERP members, and almost 32% of non-members, experienced unemployment during follow-up. Almost 43% and 29% returned to work from unemployment, indicating that not all unemployed found a new job. During follow-up, 3% of ERP members and 2% of non-members received disability pension and 28% of ERP members elected to use the ERP scheme.

**Table 2:** The number of individuals who experience a transition (only no recurrent transitions included)

	Non-member	ERP member
Transition	N (%)	N (%)
1: W → S	418 (34.75%)	1599 (39.89%)
2: W → U	383 (31.84%)	1857 (46.32%)
3: S → W	403 (33.5%)	1577 (39.34%)
4: S → U	51 (4.24%)	171 (4.27%)
5: U → W	346 (28.76%)	1711 (42.68%)
6: U → S	60 (4.99%)	188 (4.69%)
7: W,S,U → D	35 (2.91%)	75 (1.87%)
8: W,S,U → E	–	1136 (28.34%)

*ERP* early retirement pension scheme, *W* work, *S* sickness absence, *U* unemployment, *D* disability pension, *E* ERP

The results from the multi-state Cox-regression (Table 3) show the following significant associations for ERP non-members: Men have higher risk of unemployment, but lower risk of sickness absence from unemployment. Individuals with poor self-rated health have lower probability of return to work from sickness absence and higher risk of disability pensioning. While those with LTS in the year before baseline have a lower probability of returning to work from sickness absence or unemployment, they have a higher risk of sickness absence from unemployment and higher risk of disability pensioning. People with LTU in the previous year had a far higher risk of becoming unemployed (again), a lower chance of returning to work from sickness absence or unemployment, and higher risk of disability pension. Finally, individuals with §56 insurance had far higher risk of sickness absence but also higher chance of returning to work from LTS.

**Table 3:** Results of the Cox-proportional regression on the multi-state model

Transition		Covariate		Non-member	ERP member
From	To			HR (95% CL)	HR (95% CL)
		Gender	Female	1.00 (−)	1.00 (−)
W	S		Male	0.80 (0.45–1.42)	0.86 (0.71–1.04)
W	U		Male	1.97 (1.29–3.01)*	1.55 (1.26–1.91)*
S	W		Male	1.07 (0.58–1.98)	0.77 (0.60–0.99)*
S	U		Male	0.76 (0.39–1.47)	0.83 (0.52–1.32)
U	W		Male	1.16 (0.80–1.68)	1.09 (0.90–1.31)
U	S		Male	0.36 (0.21–0.64)*	0.85 (0.56–1.31)
W,S,U	D		Male	1.29 (0.64–2.57)	0.93 (0.58–1.48)
W,S,U	E		Male		0.70 (0.62–0.79)*
		Self-rated Health	Good	1.00 (−)	1.00 (−)
W	S		Poor	1.28 (0.76–2.14)	2.20 (1.74–2.79)*
W	U		Poor	1.43 (0.80–2.55)	1.46 (1.07–1.99)*
S	W		Poor	0.57 (0.37–0.88)*	0.76 (0.57–1.01)
S	U		Poor	1.15 (0.52–2.52)	0.60 (0.35–1.04)
U	W		Poor	0.91 (0.47–1.74)	0.80 (0.61–1.06)
U	S		Poor	0.54 (0.23–1.26)	0.91 (0.56–1.47)
W,S,U	D		Poor	5.48 (2.60–11.52)*	6.95 (4.21–11.46)*
W,S,U	E		Poor		1.65 (1.32–2.06)*
		LTS	No	1.00 (−)	1.00 (−)
W	S		Yes	0.97 (0.61–1.54)	1.52 (1.15–2.01)*
W	U		Yes	1.63 (0.94–2.81)	1.38 (1.04–1.82)*
S	W		Yes	0.36 (0.23–0.57)*	0.78 (0.57–1.08)
S	U		Yes	0.57 (0.28–1.19)	0.62 (0.37–1.04)
U	W		Yes	0.46 (0.26–0.83)*	0.59 (0.46–0.76)*
U	S		Yes	2.79 (1.36–5.73)*	1.07 (0.68–1.67)
W,S,U	D		Yes	4.84 (2.11–11.10)*	8.22 (4.89–13.81)*
W,S,U	E		Yes		1.54 (1.29–1.83)*
		LTU	No	1.00 (−)	1.00 (−)
W	S		Yes	0.64 (0.21–1.94)	1.42 (1.00–2.03)
W	U		Yes	40.17 (28.43–56.75)*	19.79 (16.27–24.09)*
S	W		Yes	0.33 (0.17–0.64)*	0.49 (0.32–0.74)*
S	U		Yes	12.11 (5.85–25.07)*	13.54 (8.91–20.59)*
U	W		Yes	0.37 (0.26–0.53)*	0.45 (0.38–0.53)*
U	S		Yes	1.89 (0.83–4.29)	1.16 (0.79–1.68)
W,S,U	D		Yes	3.41 (1.41–8.20)*	2.04 (1.18–3.52)*
W,S,U	E		Yes		2.05 (1.65–2.56)*
		Chronic disease §56	No	1.00 (−)	1.00 (−)
W	S		Yes	43.97 (21.09–91.69)*	17.27 (12.78–23.34)*
W	U		Yes	1.00 (0.41–2.43)	0.73 (0.40–1.34)
S	W		Yes	4.92 (2.50–9.68)*	7.98 (5.72–11.13)*
S	U		Yes	1.07 (0.32–3.58)	0.78 (0.27–2.27)
U	W		Yes	1.99 (0.73–5.41)	1.81 (1.04–3.15)*
U	S		Yes	6.07 (1.10–33.47)*	4.43 (1.99–9.85)*
W,S,U	D		Yes	1.05 (0.19–5.81)	1.05 (0.41–2.66)
W,S,U	E		Yes		0.96 (0.63–1.48)

*ERP* early retirement pension scheme, *W* work, *S* sickness absence, *U* unemployment, *D* disability pension, *E* ERP, *LTS* long term sickness absence, *LTU* long term unemployment

*: = *p* < 0.05

Among ERP members, the following significant associations were found (Table 3): Men have a higher risk of becoming unemployed, a lower probability of returning to work from sickness absence, and a lower probability of using their ERP scheme. Poor self-rated health is a significant risk factor for sickness absence, unemployment, disability pension, and early retirement using the ERP scheme. People with previous LTS have significantly higher risk of (repeated) sickness absence and unemployment, and they have significantly lower probability of returning to work from unemployment. Further, they have a higher risk of disability pension and a higher probability of using their ERP scheme. People with LTU in the previous year have a far higher risk of becoming unemployed (again), a lower chance of returning to work from sickness absence or unemployment, a higher risk of disability pension, and a higher probability of using their ERP scheme. Finally, individuals with §56 insurance had a far higher risk of sickness absence, but also a higher chance of returning to work from LTS or unemployment.

The two charts in Fig. 2 show the results from the MSLT method and the Cox-MSLT method for estimating the WLE (only work state duration). A new transition probability has been calculated for each possible age on the horizontal axis – this means that the curves express the expected duration of time spent in the work state with the only condition being that the person is in the work state at the current age. The expected duration of years spent in the work state until retirement age is shown on the vertical axis. Separate curves are shown for combinations of poor and good self-rated health and ERP members or non-members.

A comparison of the two charts in Fig. 2 illustrates the overall agreement in the expected work duration for the two methods. However, the Cox-MSLT method provides smaller confidence intervals for the small groups. Also, the Cox-MSLT method gives estimates of work duration that are lower than the estimates for the MSLT method in the age range of 55–57 years. The largest difference between the two charts is the curve for ERP non-members with poor health. Since this is the smallest of the four groups, the curves show more fluctuation and large confidence limits.

**Fig. 3:**
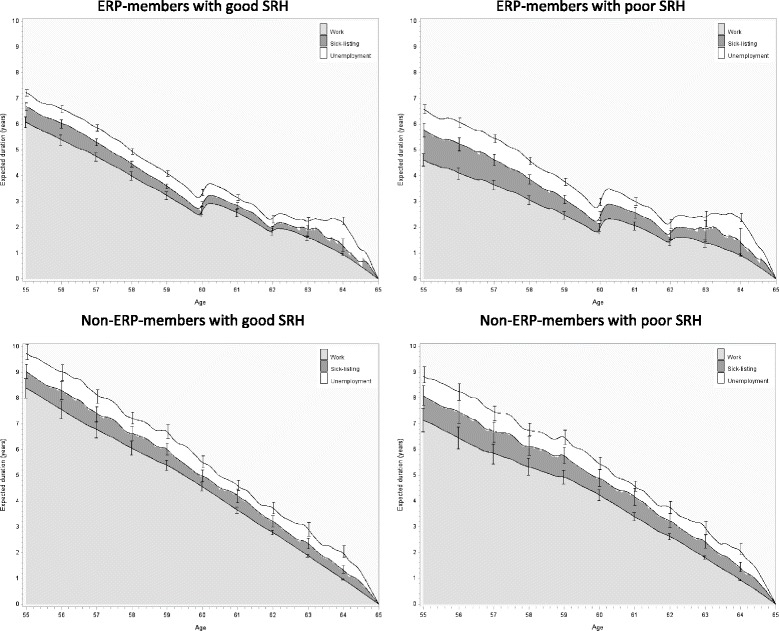
The WLE (divided by time of; work, sickness absence and unemployment) with 95% confidence limits, for the causal effect of good or poor self-rated health divided by ERP members and non-members

Figure 3 shows the estimated duration of time a person at the given age will spend in work, sickness absence or unemployment until he or she reaches retirement age (65). For example, a 55 year old ERP member reporting good health can expect to spend 7 years in the labor market, of which he or she on average will be on LTS for approximately 7 months and unemployed for six to 7 months. ERP members reporting poor self-rated health will on average spend 14 months on LTS and 10 months unemployed (Fig. 3 and Table 4). The charts also show differences in WLE between ERP members and non-members – even if both groups are in good health. The difference between members and non-members in good health is almost 2.6 years (7.21 (6.07 + 0.60 + 0.54) years for ERP members against 9.79 (8.38 + 0.66 + 0.75) years for non-members). However, the expected amount of time on sickness absence and/or unemployment is nearly the same for ERP members and non-members. In comparison, the WLE difference between good and poor health for 55 year old employees was approximately 1.4 years.

**Table 4:** Expected time (in years) working including 95% confidence limits, on long term sick leave, and unemployed for employees 55 years old

ERP	Self-rated health	Work	Sickness absence	Unemployment
		Years (95% CL)	Years (95% CL)	Years (95% CL)
Non-member	Poor	7.14 (6.67–7.60)	0.95 (0.57–1.34)	0.81 (0.49–1.12)
	Good	8.38 (8.00–8.76)	0.66 (0.38–0.95)	0.75 (0.45–1.05)
Member	Poor	4.61 (4.38–4.84)	1.15 (0.90–1.41)	0.82 (0.65–0.99)
	Good	6.07 (5.87–6.28)	0.60 (0.45–0.75)	0.54 (0.42–0.67)

*ERP* early retirement pension scheme

Due to large computational demands, the 95% upper and lower limits of the expected durations are only shown for the whole ages. The upper and lower limit of the expected duration should be used with caution, because the outcomes are dependent.

## Discussion

Using a multi-state approach to measure labor market affiliation has shown several advantages over classical analysis of single outcomes. The multi-state approach has provided valuable new knowledge for researching long-term sickness absence [3–5, 18, 22], effect of interventions regarding lower back pain [2], breast cancer [23], thyroid diseases [24], and rheumatoid arthritis [25]. Estimates of WLE provide the researcher with the means to summarize the results of a complex multi-state model in a very efficient way.

The present article compares results using a MSLT approach and a Cox-MSLT approach when calculating the WLE for a cohort of Danes. The MSLT method has shown to be a good approximation for WLE estimations [8], compared to prior methods which relied on yearly statistics of labor market affiliations and death rates [7]. The main reasons for the appeal of the MSLT method are the access to more detailed register data on labor market affiliation and death statistics as well as the significant increase in computer power. The same conditions allow the use of the Cox-MSLT method.

The Cox-MSLT method has several advantages compared to the existing MSLT method – specifically the ability to make WLE estimates for small groups by relying on the proportionality assumption. If, however, the proportionality is not valid, the Cox-MSLT will not provide good estimates, making it vital to check the assumption by visually checking the proportionality through cumulative hazard charts of each of the covariates used. The computational time of using either method is approximately the same. However, if the WLE needs to be shown for all covariates, the Cox-MSLT will be quicker because the baseline hazard is only estimated once, whereas the MSLT approach relies on a hazard curve for each combination of covariates. For both methods, the computation of confidence intervals is very time consuming even when using a powerful computer. For this reason, we only calculated confidence intervals for a few points. Further, the current calculations are simplified since they only consider the variability of the baseline hazard and do not include the variability of the individual parameter estimates.

The analysed example showed shorter WLE for people reporting poor health compared to people in good health, and for members of the ERP scheme compared to non-members. The intention of the ERP scheme is to allow early retirement for people with poor health and limited work ability. Thus, the short WLE of ERP-members with poor health is in line with the intension of the ERP scheme. However, we found that from age 57, ERP members in good health have shorter WLE than non-members in poor health. Thus, the economic possibility for early retirement also seems to be an incentive for employees in good health. As part of the Danish efforts to increase labor market participation, the ERP scheme is gradually being phased out. While this change will undoubtedly increase WLE, it has been hypothesized that lack of ERP membership would force individuals in poor health to use more sick leave or put them a higher risk of unemployment. While people in poor health can be expected to be unemployed or on sickness absence for a longer time than employees in good health, our analyses do not show that ERP non-members spend more time on unemployment or sickness absence benefit than members. However, it is possible that ERP members may be in poorer health than non-members, thus confounding the comparison of groups. While comparison of self-rated health and previous long-term sick leave shows only minor differences between ERP member and non-members it is possible that these measures do not capture all aspects of health. Therefore, residual confounding may impact the comparison of ERP members and non-members.

The relevance of estimating the WLE for members and non-members of the ERP scheme is restricted to the context of the Danish labor market. The results concerning SHR may contain some relevance to other countries, which have a labor market system comparable to the Danish system. Likewise may the multi-state approach, in which the WLE is distributed between work, unemployment and sickness absence, be relevant for countries in which it is possible to make such distinction. The relevance of estimating WLE for subgroups is high, in particular if the size of the subgroup suggest that the WLE could benefit of making assumptions about the baseline hazard.

The high level and accurate WLE estimation done in the present paper, highly relies on detailed Danish register data available. This includes information on labor market affiliation, and the possibility of linking register data with surveys through the social security number. For other countries it may be difficult to gain access to the same level of data.

The choice of SRH as explanatory variable was useful to illustrate the methodology, but also caused some restrictions. We had to limit ourselves to a sample where self-report health data was available. The limited sample size precluded the analysis of gender specific trajectories or the estimation of WLE for immigrants. WLE according to gender and migration background are important topics which could be explored in analyses based solely on register data. Research on occupational exposures would require large-scale surveys or used of register based job classification combined with job-exposure matrices.

The interpretation of WLE results depend on the assumptions behind the statistical model used to estimate WLE. If the model is seen as predictive, the WLE estimates represent the expectations for each subgroup in the model. If the model is assumed to represent causal relations, the WLE estimates represent the expected consequences of the hypothetical intervention studied. The present example should be interpreted as predictive since the data does not provide for any causal claims. In this situation, the usual caveats concerning the interpretation of causal effect from observational studies apply: ie, the risk of unmeasured confounding or that the results are specific to a subgroup that is not representative of the population of interest. The present example did not include important covariates such as education and prior long-term labor market affiliation. Also, the analysis relies on a single measurement of self-rated health, ignoring potential changes in health after baseline. The analysis could have benefited from other register data on, for example, hospitalization to track the health of individuals. Finally, the multi-state model could be expanded by including more states: eg, distinguishing been types of unemployment and between full time and part time sick leave. Thus, while the results are clear, they serve primarily to illustrate the methodology.

## Conclusion

As more details are added to register data, the statistical model used to analyze the data must keep up to utilize the potential of the enrichment. The combination of a Cox regression and a multi-state model has already proven to be a strong combination for measuring differences in labor market affiliation according to different exposures or interventions. The estimation of WLE is a natural expansion of this research, and an effective way to summarize data on labor market affiliation.

## Abbreviations

CEM: Causal effect model
CPR: Cox proportional regression
LMA: Labor market affiliation
MSLT: Multi-state life table
MSM: Multi-state model
SRH: Self-rated health
WLE: Work life expectancy
